# Influenza Vaccination: Effectiveness, Indications, and Limits in the Pediatric Population

**DOI:** 10.3389/fped.2019.00317

**Published:** 2019-07-30

**Authors:** Chiara Mameli, Ilaria Cocchi, Mara Fumagalli, Gianvincenzo Zuccotti

**Affiliations:** Department of Pediatrics, V. Buzzi Childrens' Hospital, University of Milan, Milan, Italy

**Keywords:** influenza vaccine, influenza, children, effectiveness, pediatric population

## Abstract

Influenza vaccine is considered the most effective way to prevent influenza. Nonetheless, every year vaccine coverage is lower than recommended in the pediatric population. Many factors are supposed to contribute to this phenomenon such as the uncertainty about the indication for vaccination, and the suboptimal vaccine-effectiveness in pediatric age, especially in the youngest children. In this review we discuss the effectiveness, indications, and limits of influenza vaccination in the pediatric population based on the most recent evidences.

## Introduction

Human influenza disease is primarily the result of the infection with influenza A and influenza B viruses. Type A virus can be classified using surface antigens hemagglutinin and neuraminidase. In the last years, the main circulating A strains have been the H1N1 pandemic strain and the H3N2 strain (1). The two major antigenically B viruslineages are B/Victoria and B/Yamagata (2). Each year both B virus strains co-circulate and are responsible for about 25% of influenza disease cases (3). However, the proportion of circulating influenza B strains varies by season and countries (4–6) Different strains have different virulence and prefer to infect different age clusters, probably based on previous exposure to antigenically similar viruses (1). In particular, influenza B virus is likely to affect children, and young adults. Children hospitalizations and fatal influenza cases are mainly associated with B subtypes (1).

Each year, 15–45% of children are infected with an influenza virus and by the age of 6 most children have been infected with influenza virus at least once (7). Viremic titers in children are higher than in adults and shedding of virus goes on for longer periods (8, 9). Therefore, children represent a critical source in the transmission of influenza and sustain annual epidemics (9).

About 870,000 children aged <5 years and about 300,000 children aged <1 year are hospitalized each year all over the world because of influenza and 10–15% of children need medical care for influenza-related diseases (9, 10). It is estimated that between 28,000 and 111,500 children below 5 years of age die each year due to influenza-related causes, most of them in developing countries (9–11) and from 2009, in 8/9 seasons, influenza disease course was reported to be moderate to severe in pediatric population (11).

It is unquestionable that influenza vaccine (IV) is the most effective way to prevent influenza (2). The first vaccine, a live-attenuated monovalent vaccine containing virus A, was developed immediately after the isolation of influenza virus in 1933. Fortunately, during the last decades, much has changed in the prevention of influenza in terms of vaccine manufacture, type of vaccines and strain coverage. Nowadays, two types of IVs are mainly used: an inactivated (IIV), and a live attenuated one (LAIV). The IIV can contain three or four virus strains. The trivalent ones (IIV3) are currently targeted against a H1N1 virus, a H3N2 virus, and a B virus, while the quadrivalent ones (IIV4) are targeted against both viruses A, and both viruses B. IIV3 are currently available in three different formulations: whole-virus vaccines, split-virus vaccines, and subunit vaccines. Two IIV4 vaccines have been developed and marked: a split inactivated vaccine and a subunit one. There are also adjuvanted seasonal IVs, but in most countries, they are not still licensed for children use. The LAIV is a quadrivalent vaccine containing both viruses A and B (12–14).

In this review we discuss the effectiveness, indications, limits, and ongoing direction of IVs in the pediatric population based one the most recent evidences.

## Indications of Influenza Vaccination

Although seasonal IVs are available since many decades and recommended since 1960, the first practical indications for children immunization were issued in the early two thousand and only for children with high risk conditions.

After 50 years from the first release, the benefit of the vaccination in children with chronic disorders remains unquestioned. However, the vaccination strategy, which is based only on the direct protection of those subjects at highest risk, has not been proven to be very effective in reducing influenza morbidity, and mortality, as well as not being cost-effective (15). Moreover, children, healthy or chronically ill, –especially those younger than 5 years, are at higher risk for serious influenza-related complications, such as bacterial co-infections (e.g., *S. pneumonia*e or *S. aureus*), seizures, influenza-associated encephalitis/encephalopathies, and fulminant myocarditis and pericarditis (16–22).

The extension of the seasonal IV program to all children aims to reduce the public health impact of influenza by providing direct protection and also lowering transmission rates (23). Reducing influenza transmission in the community will avert many cases of severe influenza and influenza-related deaths in older adults and in people with clinical risk factors. Additionally, from an economical perspective, IV was shown to be cost-effective for children in all analyses (24, 25).

Different types of recommendations have been released worldwide. The World Health Organization (WHO) recommends annual vaccination, prioritizing high risk groups including pregnant women, children under 5 years of age, the elderly, and those with underlying health conditions (26).

In the United States (US) the Advisory Committee on Immunization Practices included healthy children aged 6–23 months in the target population of influenza vaccine for the first time in 2004 and it included all healthy children aged more than 6 months only in 2010 (27, 28).

Many countries suggest vaccinating children older than 6 months of age with high-risk conditions only; just few ones universally recommend the vaccination for the whole pediatric population, from 6 months of age, and offer it for free till the age of 5 years (e.g., Canada, Australia) (13, 29, 30). In Europe, the European Center for Disease Control and Prevention (ECDC) has limited power over national IV policies. Therefore, each country establishes its own strategies in recommending vaccination. Not all member states have a formal national action plan for vaccination and in most countries recommendations for seasonal IV only include target or at-risk groups. During the 2017–18 influenza season, only 6/30 states recommended seasonal IVs to healthy children or adolescents (31–33).

Several reasons may explain these different immunization policies: first of all the availability of economic resources. Implementation of resources allocated to influenza vaccination is not always considered a priority for the National Health Authorities. Several economic evaluations are important to assist policymakers defining the costs of influenza vaccination programs and their financial cost-effectiveness. Secondly, influenza vaccination requires additional work that should be efficiently organized at the light of each national immunization schedules: this is of outstanding importance for the success of the influenza vaccine campaign. Despite the awareness of these economical and organizational barriers, in our opinion, the extension of the recommendation for the whole pediatric age could confer great benefits in terms of social equality.

ECDC, Centers for Disease Control and Prevention (CDC), and American Academy of Pediatrics (AAP) indicate that inactivated vaccines should be the primary choice for all children older than 6 months. However, they do not indicate which one between IIV3 and IIV4 should be preferred. Indeed, the type of the formulation is currently debated considering that the prevalence of B viruses is relatively low and varies between seasons (17, 20, 33). LAIV was not recommended in any setting in the past two influenza seasons based on data demonstrating low effectiveness against influenza A(H1N1) (34, 35). However, for the 2018–2019 influenza season, the AAP reintroduced the use of LAIV for healthy children aged older than 2 years who would not otherwise receive an influenza vaccine (13, 35). AAP supports the use of LAIV with the aim of achieving the best vaccination coverage and optimal protection in children of all ages (13).

Concerning IV's schedule, IV should be repeated every year, as recent studies suggested that there was no strong evidence of protection extended for more than one influenza season and vaccine effectiveness seems not to diminish with frequent vaccinations (36–42).

## Influenza VACCINE Effectiveness in Pediatric Age

**Figure 1:**
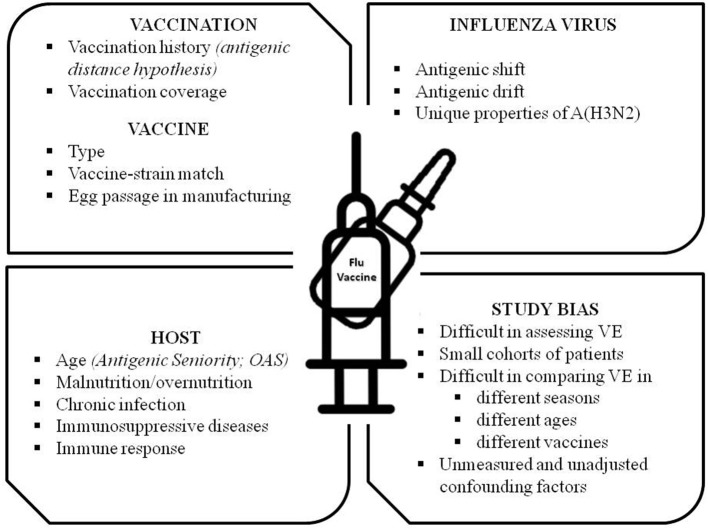
Factors and conditions affecting Influenza Vaccine Effectiveness (VE). OAS, Original Antigenic Sin.

An interesting argument of debate is the vaccine effectiveness (VE) of the available IV. As randomized controlled trials are not suitable for monitoring VE across the seasons, the test-negative design (TND), a modified case-control study, has been introduced since 2004 (43, 44). Based on the results of TND studies, the VE appears to vary from season to season, by age group, with vaccination history, and by country. Many theories and factors have been proposed over the years to explain these discrepancies, such as the suboptimal vaccine-strain match, the different types of vaccine (inactivated vs. LAIV), the vaccine manufacturing (e.g., generating egg-induced mutations in the hemagglutinin that affect antigenicity), the age-dependent patterns in protection (e.g., “Original Antigenic Sin”–*OAS*–and the more recent model of OAS: the “Antigenic Seniority”), the nutritional status, the unresponsiveness of some hosts to influenza vaccine, the vaccination coverage rates in the community, the prior influenza vaccination (e.g., “the antigenic distance hypothesis,” a theoretical framework explaining the variable effect of repeated vaccination) and the difficulty of measuring VE accurately (45–54). A list of possible factors affecting VE is reported in Figure 1. The contribution and the relative importance of each factor in determining the VE is largely unknown and it is an intriguing field of future research.

In addition, combining and interpreting differences in VE estimates from available studies is extremely challenging because VE is assessed annually (due to the frequently changing vaccine) and because of the difference in study designs, age of recruited children, influenza seasons, and countries where the studies were conducted.

Given these observations, it is extremely difficult to give data about VE in pediatric age. However, to the best of our knowledge, we summarized the available data as follows:

- Children older than 2 years: trivalent inactivated IV showed a higher VE against A/H1N1pdm09 (up to 70%) when compared to LAIV (up to 39%). However, they had similar effectiveness against influenza A/H3N2 and B (55, 56). Quadrivalent inactivated subunit-antigen vaccine showed a VE in preventing influenza illness ranging from 45 to 65% against any type of influenza, 51–71% against influenza A, and 32–34% against B. According to some authors, for this vaccine the VE seems to be highest in the younger children aged 1–5 years old (57–59).
- Children between 6 months and 2 years: there are few studies specifically assessing the VE of the inactivated vaccines between 6 and 24 months of age. The great majority of studies performed efficacy analysis or VE pooled analysis considering most often children aged from 2 to 59 months or from 2 to 7–9 years old. Based on the available data, VE in children from 6 to 24 months of age range from 18 to 85% for trivalent inactivated vaccine (60–64).

Efficacy studies showed that the recently quadrivalent split-virion inactivated vaccine was effective against influenza vaccine-like strains (50.9% efficacy against any A or B type and 68.4% against influenza caused by vaccine-like strains) in children aged 6–35 months (65). VE data are not currently available, however effectiveness is expected to be similar to those reported in efficacy studies.

## Limits of Influenza Vaccination in Pediatric Age

One of the major limits of IV in pediatric age is the absence of recommendation in infants younger than 6 months of age. The younger infants have a greater risk of severe influenza infection and a higher rate of hospitalization than older infants (66). The risk is even greater if they have chronic conditions (10). Up to now, IV has not been approved by regulators for use in infants and its use is currently off-label and arbitrary. Apart from the well-known cocoon strategy which is extremely difficult to perform, maternal immunization during pregnancy could overcome this limit and is the only measure approved by the health authorities (67). In this field, evidences about immunogenicity and efficacy are rapidly accumulating. IIV or A(H1N1) MF59-adjuvanted vaccine during pregnancy result in transplacental transfer of the generated antibodies (68). The efficacy of pregnant women's vaccination in preventing the disease and influenza-related hospitalization in infants was estimated to be around 50–60% according to different studies (69–71). However, several questions remain unanswered as which should be the best time for maternal immunization to ensure the best and the longest protection for the newborn and young infants and which is the immunological role of breastfeeding. In particular some authors reported the absence of protective antibody levels at birth and limited immunological and clinical protection up to the 3rd month of life (72, 73). Others showed that clinical protection against influenza and influenza–associated pneumonia persists up to the 6th month of life (74, 75). Considering the available data, determining acquired protection duration is imprecise, with few immunological, and effectiveness data between the third and 6th months of infant life. Moreover, long-term adverse effects of maternal immunization on infants have not been reported, and more safety studies are needed. Nonetheless, maternal immunization remains the best practice for protecting children against influenza in the 1st months of life, and should be encouraged.

Another potential limit of IV in pediatric age is the need for 2 shots in the younger naïve children, especially in infants who have a very full immunization schedule. Studies showed that the second dose was not always received or delayed far beyond the recommended interval of 28 days (76, 77). There were probably several reasons for incomplete vaccinations, such as schedule complexity, in between-doses frequent infections, difficulties in scheduling a doctor appointment, financial barriers, and lack of provider–parent discussions on the importance of the second dose (78). Efforts should be made in order to overcome the two shots with just one effective single dose for every age. This change can potentially simplify IV procedures and improve the adherence to IV schedule.

The IVs remain the primary choice for all children, even though LAIV, besides its capacity of inducing mucosal IgA antibodies, providing protection at the site of viral entry against subsequent infection, and eliciting both humoral, and cellular immune responses, may also improve the compliance thanks to its non-invasive administration (endonasal spray) (79).

## Ongoing Discussion and Future Perspectives

The characteristics of the immune system in young children on one side and the presence of an immunosuppressive disease on the other have shown to affect IV response (12, 80, 81). This raised debates about the best approach to enhance the immune response to IV in these two specific different groups. Some strategies have been tested, such as the use of higher doses of antigen, and adjuvants (82). In 2009 the high-dose (containing four times as much hemagglutinin as in standard-dose vaccines) trivalent inactivated IV was licensed for use in the elderly on the basis of its safety profile and superior immunogenicity (83). In pediatric population, recent studies showed that the high-dose IV was more immunogenic than the standard one in children with leukemia or solid tumors and in solid organ transplant patients but not in children with HIV (84), with good reported safety profile (84–86). Given the relatively small studied population, despite the evidences that immunocompromised children generate a lower immune response to standard-dose IV compared to healthy subjects (87), no definitive recommendation about the use of the high-dose IV can be drawn. Notably, no data about the use of this high dose IV are available in immunocompromised children, and younger than 2 years. Further studies are needed.

Another approach to enhance the immune response was the use of oil-in-water adjuvant MF59®, firstly approved in 1990 for adults older than 65 years of age. In 2018, a first trial assessed the relative efficacy, immunogenicity, and safety of an MF59-adjuvanted quadrivalent inactivated subunit IV (aQIV) compared to a US-licensed non-adjuvanted influenza vaccine in a large cohort of children aged between 6 months and 5 years in 2 consecutive seasons (88). The authors showed that in the youngest children (6–23 months) aQIV provided greater protection against influenza than a non-adjuvanted vaccine. The clinical benefit was demonstrated since the first vaccination in vaccine-naïve children. The efficacy and vaccine safety profiles of aQIV were similar to the non-adjuvanted comparator vaccine, with the exception of major Solicited Adverse Events. Recently, Daily and colleagues assessed the impact of repeated vaccination on immunogenicity and safety of aQIV in children aged between 6 months to 5 years. This study confirmed an enhanced immunogenicity and a similar safety profile after repeated aQIV vaccination compared to repeated non-adjuvanted influenza vaccination (89). Given the promising results, if confirmed in the ongoing trials, aQIV could be a valid option for the routine use in pediatric population in the near future.

A quadrivalent recombinant vaccine, currently available in adults, was recently studied in children (6–17 years old), and it was found to be comparable to the IIV in terms of safety, and immunogenicity (90).

The Committee for Medicinal Products for Human Use of the European Medicines Agency and the European Commission (in October 2018 and in January 2019 respectively) approved the inactivated cell-based quadrivalent influenza vaccine (QIVc) for the use in patients older than 9 years. The QIVc is supposed to be used as early as in the next influenza season (2019–2020). The different production process of the vaccine (cell-based vaccine vs. embryonated chicken eggs) represent a step forward in avoiding egg-adapted changes, vast amount of eggs, and long manufacturing time, with a comparative or even better efficacy rate (91).

## Conclusions

Influenza immunization is the best available strategy to reduce influenza-related morbidity, and mortality and virus spread. Nonetheless, questions and limits about influenza vaccine in pediatric population remain open (Table 1). Firstly, the vaccine effectiveness in children is variable and suboptimal, with reported differences according to vaccine types, seasons, and child age. Estimating the mean effectiveness remains challenging. Secondly, influenza vaccine is currently the only vaccine requiring yearly immunization, with two shots in naïve children which could influence the vaccine uptake especially in younger children. Thirdly, there is no influenza vaccine that directly protects infants <6 months of age. The most promising strategy to protect children that are too young to be vaccinated is the maternal immunization, with estimated efficacy of 50–60%. Finally, the promising recombinant, adjuvanted, and high-dose vaccines are still not universally approved in pediatric population. Addressing these issues, together with better understanding the complex immune responses induced by natural influenza infection, will be of outstanding importance to finally design future universal vaccine.

**Table 1:** Visual summary.

**Topics**	**Conclusion**
Influenza in children	Beside A strains (H1N1/H3N2), B/Victoria, and B/Yamagata circulate worldwide and are responsible for about 25% of influenza cases, especially in children. 15–45% of children are inflected with an influenza virus yearly 870,000 children <5 years are hospitalized and more than 100,000 children die every year Children with chronic disease and also the healthy ones, of any age, but especially those younger than 5 years, are at higher risk for serious influenza-related complications
Indications of influenza vaccination	Different types of recommendations have been released worldwide The main differences regard: (a) risk category (chronic conditions vs. healthy children); (b) age limit (under 5 years of age vs. the whole pediatric age vs. specific limits decided by local health authorities); (c) type of inactivated vaccine (no preference vs. quadrivalent vaccine); (d) inclusion of the LAIV
Influenza vaccine effectiveness (VE) in pediatric age	VE varies from season to season, by age group, with vaccination history and by country Combining and interpreting differences in VE estimates is challenging Children older than 2 years*; (a) trivalent inactivated vaccine: VE up to 70% against A/H1N1pdm09 (vs. up to 39% for LAIV). VE against influenza A/H3N2 and B strains similar to LAIV; (b) Quadrivalent inactivated subunit-antigen vaccine: estimated VE 45–65% against any type of influenza, 51–71% against influenza A, and 32–34% against B. Children between 6 months and 2 years*: estimated VE from 18 to 85% for trivalent inactivated vaccine. No VE data available for quadrivalent formulation.
Limits of influenza vaccination in pediatric age	The use of vaccine in infants <6 months is off- label. The efficacy of maternal immunization in preventing influenza disease in infant <6 months is estimated up to 60%. The need for 2 shots in the younger naïve children
Ongoing discussion and future perspectives	The need to find the best strategy to enhance the immune response in younger and immunocompromised children Few studies are available about high dose or adjuvanted formulation New approaches to the development of influenza vaccines are investigated

* *Data to be interpret with caution. Please, see the limits discussed into the main manuscript. LAIV, Live-attenuated intranasal vaccine; VE, vaccine effectiveness*.

## Author Contributions

CM, IC, and MF wrote the paper. GZ revised the manuscript. All authors read and approved the final manuscript.

### Conflict of Interest Statement

The authors declare that the research was conducted in the absence of any commercial or financial relationships that could be construed as a potential conflict of interest.
